# Combined laparoscopic–endoscopic resection of a bleeding giant duodenal Brunner’s gland hamartoma

**DOI:** 10.1055/a-2134-9639

**Published:** 2023-08-21

**Authors:** Alessandra Marano, Marco Sacco, Lisa Marie Rorato, Stefania Caronna, Fabrizia Di Giovanni, Mauro Santarelli, Claudio Giovanni De Angelis

**Affiliations:** 1 General and Specialist Surgery Department, Emergency General Surgery Unit, Azienda Ospedaliero Universitaria Città della Salute e della Scienza di Torino, Turin, Italy; 2 Gastroenterology Department, Endoscopy Unit, Azienda Ospedaliero Universitaria Città della Salute e della Scienza di Torino, Turin, Italy; 3 Pathology Unit, Azienda Ospedaliero Universitaria Città della Salute e della Scienza di Torino, Turin, Italy

Brunner’s gland hamartoma is a rare entity and constitutes 10.6 % of all benign duodenal tumors 1 . In symptomatic patients, endoscopy represents the first-line treatment; however, there are many technical challenges that can limit endoscopic removal, including the size and location of the lesion. Therefore, surgery may be required for complex cases 2 3 .

We present the case of a heathy 41-year-old woman who presented with melena. Upper gastrointestinal endoscopy and computed tomography scanning revealed a large polyp with ulceration on the anterior wall of the duodenal bulb ( Fig. 1 ). Endoscopic ultrasonography confirmed a hypoechoic submucosal pedunculated polyp with a 10-mm base and 50-mm head. No malignancy was revealed on biopsy.

**Fig. 1:**
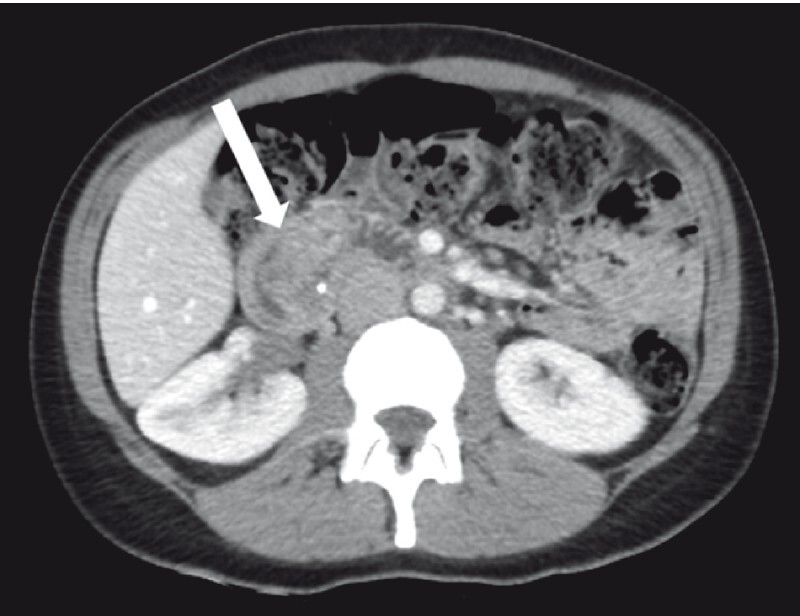
Computed tomography image showing the large duodenal polyp (arrow).

Initially, a standard polypectomy was attempted but the head of the lesion was too large to pass through the pylorus. Therefore, a combined laparoscopic–endoscopic approach was planned ( Video 1 ). During the laparoscopic exploration of the abdominal cavity, the duodenal polyp with full endophytic growth was recognized. Even with laparoscopic assistance, passage of the lesion into the stomach was not possible. Therefore, a 15-mL epinephrine solution (diluted 1:20 000) was injected into the head of the polyp to achieve volume reduction and reduce bleeding 4 . Next, the head of the lesion was pushed into the stomach by gently pressing the laparoscopic forceps along the duodenum towards the pylorus, and piecemeal resection of the head was carried out; the polypectomy was completed with the en bloc removal of the peduncle and all of the fragments were collected ( Fig. 2 ).

**Video 1** Laparoscopic-assisted polypectomy of the giant Brunner’s gland hamartoma.


**Fig. 2:**
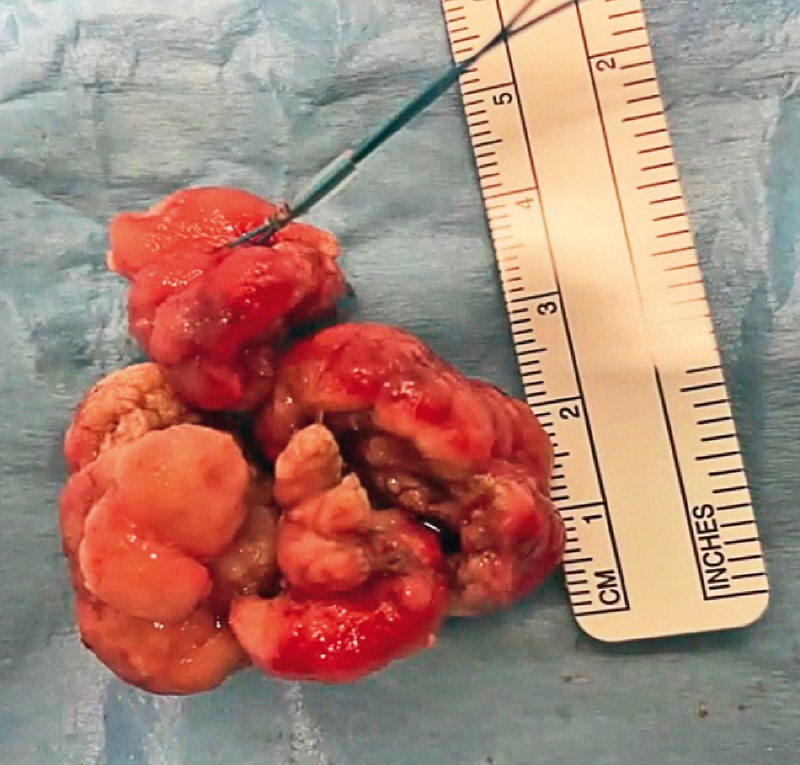
Macroscopic appearance of the specimen, which measured 50 × 35 × 15 mm.

The total operative time was 80 minutes. The patient’s postoperative course was uneventful. Pathology confirmed the lesion was a duodenal Brunner’s gland hamartoma ( Fig. 3 ). No recurrence was detected at the 6-month follow-up endoscopy.

**Fig. 3:**
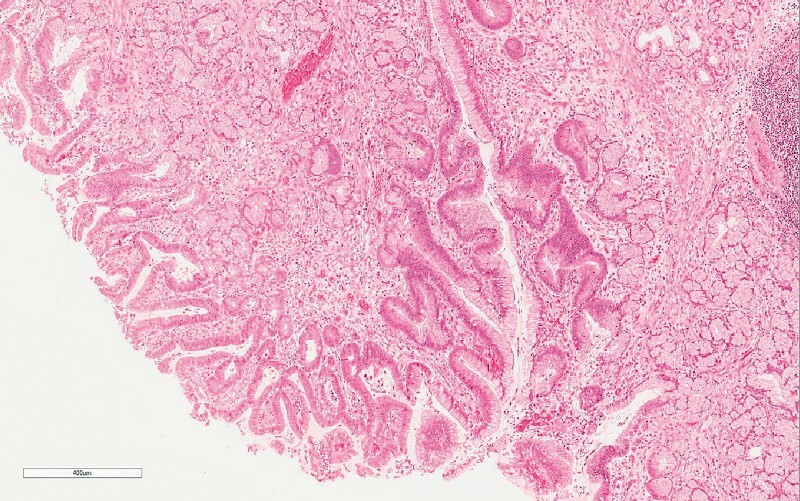
Microscopic section stained with hematoxylin and eosin (magnification × 10) showing a lining epithelium of atypical cells (probably dysplastic), stromal edema, and mild vascular congestion in relation to the Brunnerʼs glands.

In the present case, the application of laparoscopy overcame the polyp size-related constraints, allowing endoscopic resection; the combined approach provided a safe and curative therapeutic strategy, avoiding a more invasive surgical treatment.

Endoscopy_UCTN_Code_CPL_1AH_2AZ

## Notes

Endoscopy E-Videos

https://eref.thieme.de/e-videos
*E-Videos* is an open access online section of the journal *Endoscopy* , reporting on interesting cases and new techniques in gastroenterological endoscopy. All papers include a high-quality video and are published with a Creative Commons CC-BY license. Endoscopy E-Videos qualify for HINARI discounts and waivers and eligibility is automatically checked during the submission process. We grant 100% waivers to articles whose corresponding authors are based in Group A countries and 50% waivers to those who are based in Group B countries as classified by Research4Life (see: https://www.research4life.org/access/eligibility/ ). This section has its own submission website at https://mc.manuscriptcentral.com/e-videos
